# Is there a need for a clear advice? A retrospective comparative analysis of ethics consultations with and without recommendations in a maximum-care university hospital

**DOI:** 10.1186/s12910-021-00590-x

**Published:** 2021-03-02

**Authors:** Dagmar Schmitz, Dominik Groß, Roman Pauli

**Affiliations:** Institute for History, Theory and Ethics in Medicine, RWTH Aachen University, Wendlingweg 2, 52074 Aachen, Germany

**Keywords:** Ethics consultation, Ethics facilitation, Professional ethics

## Abstract

**Background:**

The theory and practice of ethics consultations (ECs) in health care are still characterized by many controversies, including, for example, the practice of giving recommendations. These controversies are complicated by an astonishing lack of evidence in the whole field. It is not clear how often a recommendation is issued in ethics consultations and when and why this step is taken. Especially in a facilitation model in which giving recommendations is optional, more data would be helpful to evaluate daily practice, ensure that this practice is in line with the overarching goals of this approach and support the development of standards.

**Methods:**

We analyzed all consultations requested from an EC service working under a facilitation approach at a maximum-care university hospital in Germany over a period of more than 10 years. Our aim was to better understand why—and under what circumstances—some consultation requests result in a recommendation, whereas others can be sufficiently addressed solely by facilitated meetings. We especially wanted to know when and why clients felt the need for clear advice from the EC service while in other cases they did not. We compared ethics consultations in terms of the differences between cases with and without recommendations issued by the ethics consultants using χ^2^ difference tests and Welch’s t-test.

**Results:**

A total of 243 ECs were carried out between September 2008 and December 2019. In approximately half of the cases, a recommendation was given. All recommendations were issued upon the request of clients. When physicians asked for an EC, the consultation was significantly more likely to result in a recommendation than when the EC was requested by any other party. ECs in cases on wards with ethics rounds resulted in comparably fewer recommendations than those in wards without ethics rounds. When interpersonal conflicts were part of the problem or relatives were present in the meeting, clients less frequently asked for a recommendation.

**Conclusion:**

From the client’s point of view, there does not seem to be only one “right” way to provide ethics consultations, but rather several. While facilitated meetings are obviously appreciated by clients, there also seem to be situations in which a recommendation is desired (especially by physicians). Further empirical and theoretical research is needed to validate our single-center results and re-evaluate the role of recommendations in ethics consultations.

**Supplementary Information:**

The online version contains supplementary material available at 10.1186/s12910-021-00590-x.

## Background

Ethics consultation services in hospitals are delivered by committee members or single consultants who take action in response to requests for assistance. The requesting parties (health care professionals, patients, relatives) may have experienced uncertainties or conflict in decision-making processes concerning an ethically best course of action in clinical practice [1]. The theory and practice of ethics consultations (ECs) in health care are still characterized by many controversies, including, for example, the adequate approach to consultation (such as the clinical vs facilitation model) or which professional group should serve in these models (such as clinicians vs nonclinicians) [2, 3]. An especially heated debate concerns the practice of giving recommendations in EC. Although there is an unhelpful lack of clarity in its definition [4], a recommendation in the context of EC is typically expected to indicate an ethically best course of action [5] that is either content-heavy (e.g., “withhold further resuscitation measures”) or process-heavy (e.g., “contact the absent child of the patient to determine more about her preferences and values”) [6]. While some authors see it as the main task of ethics consultants to offer “suggestions that improve the process and outcome of patients’ care” [2], others state that this conventional “clinical” approach to ethics consultations, which typically results in a suggestion, recommendation or advice, is outdated and should be replaced by alternative models, such as those proposed in bioethics mediation [7]. One of the main arguments in favor of giving recommendations is that this practice is closest to clinical consultation practice and is the best way to promote “excellence in outcome for each and every patient” [8]. Critics of giving recommendations argue that it lacks justification and has negative consequences. Some, for example, state that it is unclear what kind of expertise enables an ethics consultant to offer a recommendation or advice in ethically complex situations (and thus legitimizes this offer) [9]. Other authors hold that especially in cases of conflict, a recommendation does not support a good outcome in terms of closure for the parties in conflict and often marks one party as “wrong” or “losing” in the conflict [10]. A compromise in this regard is the ethics facilitation approach, which dominates the academic debate and is favored by one of the most influential professional organizations in bioethics worldwide, the American Society for Bioethics and Humanities (ASBH) [1]. It primarily aims to facilitate a principled ethical solution developed by the clients themselves in case of value uncertainty or conflict regarding value-laden concerns but does not preclude giving recommendations in specific situations.

The described controversies are complicated by an astonishing lack of evidence in the whole field of ethics consultation. We do not know much about by whom and how EC is offered. Neither do we have sufficient knowledge about the ends and outcomes of EC [11–14]. Accordingly, it is not clear how often a recommendation is issued in ethics consultations. The few available older studies indicate that many EC services frequently involve giving recommendations [7, 14], but they have not examined when and why this step is taken. Especially in a facilitation model with optional recommendations, collecting more data would be helpful to evaluate daily practice, ensure that it is in line with the overarching goals of this approach and support the development of standards. Moreover, with respect to the very large body of critical theoretical literature, it seems to be important to learn more about the premises and circumstances of giving recommendations and add some practical insight to the controversial debate.

Therefore, we analyzed all consultations of an EC service working under a facilitation approach at a maximum-care university hospital in Germany over a period of more than 10 years. Our aim was to better understand why—and under what circumstances—some consultation requests result in a recommendation, whereas others can be sufficiently addressed solely by facilitated meetings. In principle, two situations can lead to a recommendation during EC in this institution: (a) the options developed in the facilitated meeting seem ethically unsupportable from the perspective of the EC service, or (b) the requesting party asks for a recommendation from the EC service. We were especially interested in the latter: When and why do clients feel the need for clear advice from the EC service while in other cases they do not?

### Ethics consultation at a maximum-care university hospital in Germany

The Uniklinik RWTH Aachen (UKA) is a large maximum-care university hospital in western Germany with 1 400 beds and nearly 50 000 inpatients annually. Its intensive care units (ICUs) consist of interdisciplinary ICUs (103 beds), medical ICUs (36 + 14 beds) and a neurological intensive care unit for adults, as well as a neonatal/pediatric intensive care unit. In 2008, the EC service at UKA was founded as a clinical ethics committee by resolution of the management board of the hospital that thereby answered to a joint initiative of the Institute of History, Theory and Ethics of Medicine and the Clinic for Palliative Medicine (with both institutions being headed by medically trained directors).

The committee has 18–20 members (part of whom have special, certified training in ethics consultation), is multidisciplinary and initially defined consultation, education and policy work (all upon request) as its main tasks. In doing so, it primarily aimed to support patients, relatives and professionals in ethically complex conflicts and decision-making. The committee drew up its own rules of procedure in 2009, which did not address the issue of recommendation giving in ethics consultations in detail. However, it had been the common understanding of the founding members that consultations are not only aiming at “ethics facilitation” in accordance with ASBH guidelines [1] (facilitating a “principled resolution” within the boundaries of widely accepted ethical principles [15]), but can also include recommendation giving. The rules of procedure, for example, state that no committee member can be forced to participate in a consultation or recommendation. A pure facilitation approach was never intended by the committee.

The first contact for all consultation requests is a clinical ethicist (key contact person), who worked as a physician before specializing in clinical ethics and ethics consultation. She has additional training in conflict mediation. Only three members of the committee are nonclinicians (pastors and an ethicist), and the remaining members are physicians, nurses or case managers or have two qualifications (physician and ethicist). Initially, the number of consultation requests at UKA was low, as is the case in many other EC services in Germany [16] and internationally [14, 17], with 2–6 requests annually. In 2010/2011, the service implemented additional ethics rounds as a structure for continuous discussion of ethical aspects in patient-centered rounds in intensive care units. Usually, a clinical ethicist participates in these rounds—provides support based on professional expertise, if needed—and ensures that the reflection process follows given rules. In addition to the usual daily treatment plan resulting from interdisciplinary communication, during ethics rounds, the whole team explicitly reflects on ethical aspects in their discussion of each patient, including patient wishes, end-of-life issues, issues of patient autonomy, surrogate decision-making and differing perspectives among the staff when appropriate. This initiative was prompted by ICU teams, and ethics rounds were implemented in 2011. At the same time, requests for ethics consultation rose to 20–30 requests annually, with more than 80% coming from the intensive care units.

The EC service at UKA addresses consultation requests using a tiered approach and always starts with facilitation efforts in one or several meetings with all parties involved to support clarification in value uncertainties or to mediate in case of conflict. These facilitation efforts can result in four different outcomes:

1. The facilitation succeeds in the resolution of a conflict or the clarification of value uncertainties, so that the parties are able to reach a consensus regarding for example the next steps of the treatment plan or the limitation of therapy.
2. It does not succeed in developing a resolution or clarification and the clients wish to proceed with a subsequent meeting in a conventional consultation setting, where the EC service typically gives a recommendation, i.e., offers advice as to what an ethically best course of action could be.
3. The resolution or clarification developed in the facilitated meeting seem ethically unsupportable (i.e. clearly incompatible with widely accepted ethical principles) from the perspective of the EC service. Than the EC service proceeds with a subsequent meeting in a conventional consultation setting, where it typically gives a recommendation.
4. As an additional option for example in urgent cases and again typically on request of clients, a recommendation can be given at the end of the first facilitated meeting without any further conventional consultation meeting. In such cases, the committee member involved has to switch roles during the meeting (from facilitator to consultant).

Usually, three to five members of the committee (key contact person, one chairperson and one to three other members) take part in conventional consultations. Facilitated meetings, in contrast, are usually conducted by only one or two members of the committee (key contact person and a chairperson/other member).

## Methods

In line with the requirements of the Central Ethics Committee of the German Medical Association [18] and guided by the recommendations for the documentation of ethics case consultations of the working group "Ethics Advice in Hospitals" at the Academy for Ethics in Medicine [19], all consultations at UKA are documented case wise. The case documentation form used for this purpose contains all the information collected during a consultation. A template of this form is provided in the Additional file 1. The data for this study were drawn from this documentation, with a total of N = 243 consultations over more than a decade from September 2008 to December 2019.

### Boundary conditions for ethics consultation

As outlined below, prior research suggests that certain boundary conditions, different professional expectations and case-specific constellations can have an impact on clients’ wishes for recommendations by EC services. Based on these findings, we assume that four different key areas could potentially play a role here:

- Ethics consultants

As discussed by several authors, it could be a function of professional socialization, the type of training and/or the individual abilities, preferences and skills of the ethics consultant that influences whether the response to a consultation request results in a recommendation or facilitation (or the suggestion of the respective approach to the clients) [2, 3]. The team of ethics consultants at UKA worked very consistently over the analyzed period and for every kind of request, responding with the same clinical ethicist as a key contact person and facilitation efforts as a first step. Nevertheless, in some consultations, clients asked for the second (recommendation) step, whereas in others, they did not. Influencing aspects on behalf of the consultants could be the changing capabilities of the individual consultants, i.e., their growing experience or additional training. Training or experience effects could, for example, become visible over time in terms of a change in the ratio of consultations with and without recommendations.

- Requesting parties/involved agents

With regard to the requesting parties, it has been hypothesized that physicians prefer approaches to consultation requests that differ from the approaches preferred by nurses or patients’ relatives [20, 21]. In addition, varying (more or less hierarchical) communicative and decisional structures among the different medical disciplines could contribute to the preferences or expectations of medical teams as requesting parties [22, 23]. We are especially interested in possible differences in relation to the availability of ethics rounds, which had been implemented in some intensive care units. Our own previous research indicates that this instrument does have an effect on ethics consultations in our clinic [24]. Differences in the requests for recommendations by the EC service should thus be visible in the comparison of ethics consultations in the clinics and wards with or without ethics rounds.

- Nature of the request

The topic of the request (end of life, compulsory treatment, etc.) or the reliability of knowledge concerning the patient’s will could have an influence on the way clients want it to be addressed (i.e., with or without recommendation). In consultations concerning end-of-life questions, clients might more often ask for a recommendation because of the potentially grave consequences. The same might be true for requests where little or nothing is known about the patient’s will and where the basis for decision-making, therefore, might seem especially unstable. A third, possibly important characteristic in this area is whether interpersonal conflicts are (co)triggering the request for consultation. In the case of interpersonal conflict, the requesting parties could, for example, wish for a neutral third party to judge who is “right” or “wrong”.

- Process of consultation

Regarding the consultation process, the EC service at UKA has an established tiered facilitation approach, with facilitated meetings as a first step. What varies, however, is the way in which different parties are involved in the process. In some cases, medical teams wish to clarify their own way of handling a conflict with an EC service. In other cases, teams want to involve the relatives directly in the consultation process. These various settings might be connected to the way the request is addressed (with or without recommendation). We therefore examined whether consultations with and without recommendation differ with regard to the consultation setting in terms of the parties involved. Another possibly important feature is the urgency of the requests, as facilitation efforts are presumably more time-consuming than giving a recommendation.

We compared ethics consultations in terms of differences between cases with and without recommendations issued by the ethics consultants using χ^2^ difference tests and Welch’s t-test. χ^2^ difference tests allow the assessment of the statistical significance of observed relationships between two nominal scaled variables. Statistical significance supposes that an observed difference between two or more variables is large enough that the differences are unlikely to be random. We used the common test criterion of p < 0.05, with a significant test result indicating that the observed relationship was unlikely to be random, allowing for a 5-percent probability of residual error. χ^2^ difference tests do not offer any information about the strength of an observed relationship. We therefore reported Cramer’s V and interpreted effect sizes according to the standards established by Cohen [25], with V = 0.1 indicating small effects, V = 0.3 indicating medium effects and V = 0.5 indicating large effects. We assumed the EC result to vary according to the patient’s clinic or ward and according to who issued the request for an EC. We also assumed the composition of ECs as well as interpersonal conflicts between or within the team or with relatives to impact recommendation practices. To account for differences between cases with and without recommendations that may be caused by the EC composition in terms of metric variables, we used Welch’s t-test to compare the two group means for the metric variables. Welch's unequal variances t-test is an adaption of Student’s t-test; its robustness to violations of distribution assumptions is why some authors suggest to always use Welch’s t-test in preference to the Student’s t-test or Mann–Whitney U test [26]. All analyses were performed using IBM SPSS 25.

## Results

We begin our analysis with a descriptive look at key parameters of the 243 ECs at UKA between September 2008 and December 2019.

In 43.9% (n = 104) of the ECs, the patient was female, information on gender was missing in 6 cases. The patients’ ages ranged from 0 to 92 years, with a mean age of 58.6 years (standard deviation = 22.7 years). Requests were made for patients from internal (intensive) medicine (44.0%, n = 107), operative intensive medicine (21.8%, n = 53), neurology (15.2%, n = 37), weaning^1^ (6.2%, n = 15), pediatrics (5.3%, n = 13) and other wards or clinics (7.4%, n = 18).

In most of the cases (81.1%, n = 197), a physician issued the request for EC, whereas requests by relatives (9.5%, n = 23), nurses (4.9%, n = 12), multiple parties (4.1%, n = 10) or the patients themselves (0.4%; n = 1) were comparably rare. Requests were often solely processed with facilitated meetings, either with relatives (24.7%, n = 60) or the team (24.3%, n = 57). Sometimes, these formats were also combined (14.4%, n = 35), i.e., several facilitated meetings with different participants took place. Only a certain part of the requests proceeded from facilitation to a conventional consultation (35.3%, n = 83). Almost two-thirds (64.2%, n = 156) of all ECs (facilitated meetings as well as conventional consultations) involved several parties, and one-third (32.5%, n = 79) involved only the team. ECs with relatives or patients only were rather rare (3.3%, n = 8).

Many requests concerned end-of-life decisions alone (41.9%) or in combination with other topics (39.0%), whereas patient will (14.4%), ethical aspects of transplantation, termination of pregnancy and intercultural conflicts were less prominent in ethics consultations at UKA. Half of the requests required a response within 24 h (48.3%, n = 98), and the other half required a response within the course of one week or later (51.7%, n = 105). Urgent requests were typically issued in situations, where a delay in decision-making could cause additional suffering for the patient or might lead to a life-threatening situation.

For 236 of the 243 ECs from September 2008 to December 2019, it was documented whether the EC resulted in a recommendation. In seven cases, this information was missing. Categorization of the ECs based on whether a recommendation was given resulted in the data being split into approximately two equal halves: In 55.5% (n = 131) of the ECs, a recommendation was given. Table 1 provides an overview of the differing outcomes of various types of ECs.

**Table 1:** Crosstab recommendation according to type of consultation

	Formal consultation	Facilitated meeting with team	Facilitated meeting with relatives	Combined facilitated meetings	Total
**No recommendation**					
n	8	23	49	20	100
%	9.6	42.6	83.1	57.1	43.3
**Recommendation**					
n	75	31	10	15	131
%	90.4	57.4	16.9	42.9	56.7
**Total**					
n	83	54	59	35	231
%	100	100	100	100	100

N = 231; Cramer’s V = 0.59; p = 0.000

As could have been expected, conventional consultations mostly resulted in a recommendation, whereas facilitated meetings with relatives usually did not. With facilitated team meetings and combinations of several facilitated meetings (i.e., both facilitated meetings with the team and relatives), this relationship was not as evident. In summary, the overview indicates a strong relationship between the format of the discussions and the outcome of the ECs. All recommendations were requested by clients. It was never the case that ethics consultants had to change roles from facilitator to consultant because the options developed in the facilitated meeting were ethically unsupportable.

**Fig. 1:**
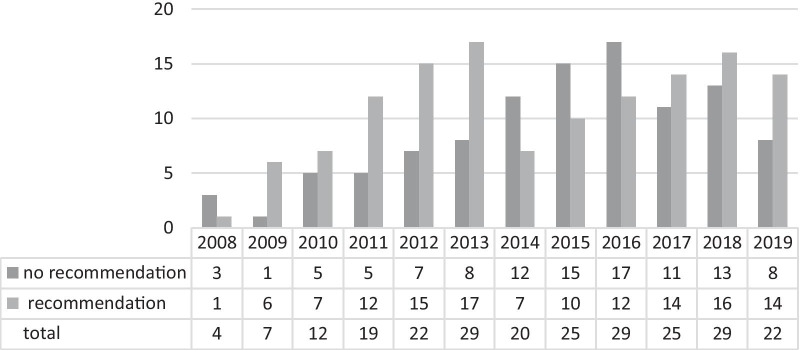
Number of total consultation requests and recommendation outcomes from 2008 to 2019. Note: Information on “recommendation or no recommendation” was not documented for two cases in 2011, four cases in 2013 and one case in 2014

Figure 1 provides an overview of the total number of ECs and EC outcomes in terms of recommendations given (2008 to 2019). After ECs were established at UKA in 2008, the demand for consultations constantly increased over the following five years, as indicated by the growing number of total consultation requests from 2009 to 2013. During this period, the proportion of consultation requests that resulted in a recommendation being issued was consistently well over 50%. In the following years, the number of consultations remained at a comparably high level, with only slight fluctuations. However, the clear pattern of a preference for giving recommendations was interrupted from 2014 to 2016.

- Ethics consultants

As stated above, the ethics consultants at UKA worked very consistently in an only marginally changed team over the analyzed period and for every kind of request, responding with the same clinical ethicist as a key contact person. Changes in recommendation patterns over time, which could potentially be related to the training or professional experience of the consultants, were not detectable with the available data.

- Requesting parties/agents involved

We tested for significant differences in the issuance of recommendations due to the requesting parties, i.e., depending on whether the request was made by physicians, nurses, the patient itself, a relative or by several of these parties. Table 2 indicates a significant difference in the issuance of recommendations depending on who issued the request. When physicians requested ECs, the consultation request was significantly more likely to result in a recommendation than with any other party.

**Table 2:** Crosstab recommendation according to requesting parties

	Physicians	Non-physicians	Total
**No recommendation**			
n	74	31	105
%	38.1	73.8	44.5
**Recommendation**			
n	120	11	131
%	61.9	26.2	55.5
**Total**			
n	194	42	236
%	100	100	100

N = 236; Cramer’s V = 0.27; p = 0.000

A significant effect is also evident with regard to the medical disciplines: requests from neurology and pediatrics more frequently resulted in recommendations by the EC service than those from any other single ward or clinic (n = 223, Cramer’s V = 0.27; p = 0.004). However, differences according to the disciplines may be at least partly attributable to the neurological and pediatric clinics not having ethics rounds, whereas other wards and clinics do. Accordingly, we compared differences in recommendations for wards with and without ethics rounds. Table 3 indicates that EC in cases on wards with ethics rounds resulted in comparably fewer recommendations than for wards without ethics rounds.

**Table 3:** Crosstab recommendation according to wards with and without ethics rounds

	Ward without ethics rounds	Ward with ethics rounds	Total
**No recommendation**			
n	53	52	105
%	36.6	57.1	44.5
**Recommendation**			
n	92	39	131
%	63.4	42.9	55.5
**Total**			
n	145	91	236
%	100	100	100

N = 236; Cramer’s V = 0.20; p = 0.002

- Nature of request

In general, the topic of the consultation request, i.e., whether the consultation concerned end-of-life questions, patient will, transplantation or termination of pregnancy, was not significantly related to differences in recommendation frequencies. Due to the small number of cases in some groups, this finding is, however, only tentative. Whether or not a recommendation was given also seemed to be independent of the existence of information concerning the patient’s will. Differences according to the availability of a living will and/or a power of attorney were not significant.

We also examined the relevance of interpersonal conflicts for EC recommendations being given. The results indicate that it does make a difference whether an interpersonal conflict exists: In the case of interpersonal conflicts, ethics consultations resulted in slightly fewer recommendations (n = 235; Cramer's V: 0.15; p = 0.017). This tendency was especially evident in conflicts within the medical team, as indicated by Table 4. Notably, due to small group sizes, we refrain from reporting test statistics when taking into account the entire range of characteristics describing possible interpersonal conflicts. It is also notable that interpersonal conflicts were part of the consultation in less than half of the EC cases (n = 109).

**Table 4:** Crosstab recommendation according to demonstrable interpersonal conflict

	Medical team/relatives	Among relatives	Among medical team	Interdisciplinary conflict	Several conflict lines	Total
**No recommendation**						
n	44	2	7	1	4	58
%	52.4	40.0	87.5	33.3	40.0	52.3
**Recommendation**						
n	40	3	1	2	6	52
%	47.6	60.0	12.5	66.7	60.0	47.7
**Total**						
n	84	5	8	3	10	110
%	100	100	100	100	100	100

N = 110

- Process of consultation

The urgency of the request, i.e., whether a request required an immediate response, was not statistically significantly related to whether a recommendation was issued. However, whether relatives were present during the meeting made a decisive difference: While almost three-quarters (72.1%) of the consultations without relatives resulted in a recommendation, this was not even the case in half (47.2%) of the discussions in which relatives were present (n = 230; Cramer’s V = 0.24; p < 0.000). In the majority of the consultations (59.3%, n = 144), relatives took part, whereas over a third of the consultations (35.8%, n = 87) took place without relatives; for approximately 5% of the consultations (n = 12), this information was missing.

## Discussion

We aimed to better understand the circumstances under which clients feel the need for clear advice in an EC. Based on the dataset of N = 243 consultations by an EC service that occurred over a period of 11 years, we are, however, limited to analytical approaches that fit the information on a small number of cases provided by process-produced data. Some analyses are not meaningful simply due to insufficient group sizes in our dataset (e.g., relating consultation outcomes to different topics of consultations). χ^2^-square-based test statistics are sensitive to the number of cases in the sense that with a larger number of cases, smaller associations are detected. Nonsignificant associations in our analyses may actually be significant (type II error). Thus, we can assume that the significant associations we found even with small case groups are in fact statistically meaningful. Each case discussed in a clinical ethics consultation is unique; i.e., a case will usually not arise several times with exactly the same ethical question. As a result, and despite a long period of data collection, all analyses in this contribution are cross-sectional, except trend statistics on the development of EC request numbers. Accordingly, the analyses in this paper do not allow for causal inference based on formal statistics but are logically reasoned. Nevertheless, the findings presented encourage critical reflection on the practice of issuing recommendation in EC.

The tiered facilitation approach at UKA and the analyzed period of time seem to be especially suited for our question: Here, in all cases, the way in which the EC service responded (with or without a recommendation) had not been predetermined by standards or decided by the EC consultant but instead had been chosen by the requesting parties themselves. Possible effects of training or experience of EC consultants on recommendation practices could not be analysed in detail in our study due to insufficient data. In view of the aforementioned logical arguments (consistent team, initial facilitation efforts), however, we assume that we can dismiss such effects. In addition, it is important to note that the perspective described in our results is mainly a physician perspective because the majority of requests at UKA had been issued by this profession. Only a small portion came from nurses, relatives or patients themselves. Nevertheless, the fact that the vast majority of inquiries came from physicians is an interesting statement per se.

We have shown that clients at UKA wanted *both* facilitation and recommendations in certain situations. This statement can be made consistently throughout the period of investigation. With regard to the repeatedly invoked “great divide”[8] between the conventional clinical (consultation) model and newer approaches based mainly on facilitation, this observation indicates that the practical relevance of this theoretical problem might not be as high as suggested by some authors. Although approaches based on facilitation according to DeRenzo have moved too far “from bedside”[8], they seem to be appreciated by all requesting parties in our institution.

Our results are in line with the assumption of a strong tendency among physicians to ask for case-related advice or a recommendation when involving an EC service. Several possible explanations for this assumed tendency among physicians come to mind. They are all linked to the specific practical knowledge of physicians, which is an integral constituent of clinical judgment and decision-making in clinical care and is passed on in medical training from one generation of physicians to the next [27]. Because all chairpersons as well as the key contact person and many other members of UKA EC service are physicians, clients might think they are expected to interact in a way similar to interactions with physicians from other clinical disciplines (e.g., in consults). It might also be the case that it is the favored way of interaction with other healthcare professionals for requesting clinicians because it goes well with their learned and established strategies of cooperation and communication, with their professional socialization and tradition. What fits into this picture is our finding that clinicians less frequently ask for recommendations when relatives are present in the meeting.

However, is a recommendation or advice from an EC service—from a physician’s point of view—not only an easy and well-known practice but also the best way to approach an ethical problem? Our results raise doubts regarding the universality of any such statement. We were able to show that clients (again, mostly clinicians) from wards with implemented ethics rounds less frequently ask for a recommendation. Ethics rounds are an instrument of clinical ethics that provides a structure for healthcare professionals from different professions and disciplines to continuously discuss ethically relevant aspects of patient care. It aims to develop relevant competencies in the healthcare professionals themselves and has effects not only on case-related decisions and actions but also on systems and processes in the hospital [24]. It could be hypothesized that implemented ethics rounds change the practical knowledge of clinicians. As a consequence, they could also change what clinicians expect and need from an EC service and how much decision-making authority they attribute to themselves—with the result that they less often need concrete advice from an ethics consultant.

## Conclusion

Our single-center study is only able to provide a couple of preliminary answers to some of the many open questions in the field of clinical ethics. While ethics facilitation was obviously appreciated, there also seemed to be situations in which a recommendation was desired (especially by physicians). Whenever interpersonal conflicts were part of the problem or relatives were present in the meeting, clients less frequently asked for a recommendation. From the point of view of clients, there does not seem to be only one “right” way to provide clinical ethics support; instead, there appear to be several.

Further empirical and theoretical research is needed to validate our single-center results. The client’s wishes cannot and should not serve as a sufficient justification for recommendation practices of a EC service, but our results seem to ask for a more differentiated perspective and also a theoretical re-evaluation of the role of recommendations in ethics consultations. It might be worth questioning not only the old unhelpful dichotomy of the clinical (consultation) model and newer facilitation-based models but also the recently promoted way out. DeRenzo, for example, argues that “after 30 years of experience (…) it is time to take the two existing models (…) and reshape and merge them into an HMCE (Hospital Model of Clinical Ethics) that takes the best of both and drops that which works least well” [8]. However, the envisioned HMCE has conspicuously much in common with the conventional consultation model. The author criticizes facilitation approaches for having their goals “not aligned with the goals of clinical care” and explicitly asks the consultant to give up impartiality and contribute recommendations. Our findings, in contrast, suggest that the facilitation models offer a more promising basis for reshaping the profession and supporting health care professionals in ethical decision-making: Although ethically complex decisions and situations are experienced frequently in everyday clinical care, the number of requests for ethics consultations is often low. This implies that many ethical conflicts and uncertainties are dealt with by health care professionals alone. If ethics rounds and ethics facilitation can help empower healthcare professionals with the competencies needed to tackle ethically complex situations in clinical care, their benefits might go beyond conventional case consultations. In the end, there might be less need for clear advice in ethics consultations.

## Supplementary Information

**Additional file 1:** Case documentation form.

## Data Availability

The datasets used and/or analyzed during the current study are available from the corresponding author on reasonable request.

## Abbreviations

ASBH: American Society for Bioethics and Humanities
EC: Ethics consultation
HMCE: Hospital model of clinical ethics
ICU: Intensive care unit
UKA: Uniklinik RWTH Aachen

## Footnotes

1 A weaning unit is a respiratory special care unit for the treatment of patients requiring prolonged mechanical ventilation.
