# Supplementary material for: Is there a need for a clear advice? A retrospective comparative analysis of ethics consultations with and without recommendations in a maximum-care university hospital
Source: BMC Med Ethics. 2021 Mar 2;22:20. doi: 10.1186/s12910-021-00590-x (PMC7927398; doi:10.1186/s12910-021-00590-x)
Supplement: Supplementary file 1 — Additional file 1: Case documentation form. [file 12910_2021_590_MOESM1_ESM.pdf]

Case Documentation

Date: ____________

Request from:

☐ physician / ☐ nursing staff / ☐ patient / ☐ patient relatives / ☐ rounds / ☐ _____________

Name:

Clinic / ward:

Phone Number:

Request received via: ☐ phone / ☐ fax / ☐ E-Mail / ☐ mail

Urgency:
☐ immediately / urgent ☐ within 24 hours
☐ within one week ☐ not urgent
☐ retrospective case review

Patient:

Name:

Sex: ☐ female / ☐ male

Age and Date of birth: ____________

Capacity for consent: ☐ yes / ☐ no / ☐ unclear

Advance directives: ☐ Living will
☐ Health care power of attorney

Patient advocate: ☐ Guardianship: ___________________________
☐ Health care power of attorney: ________________________

Topic / Conflict (Reason for request):

Meeting details:

Date: _____________

Format: ☐ case consultation / ☐ facilitated team meeting
☐ facilitated meeting with patient relatives
☐ retrospective case review

Participants:

Moderation:

Details:

Results / Recommendations:

Further course:

Recommendation implemented: ☐ yes / ☐ no

Deceased: ☐ yes (Date _______________) / ☐ no

Discharge:	☐ Rehab / weaning station		☐ care facility
	☐ hospice	☐ home	☐ transfer clinic / ward

Details:
